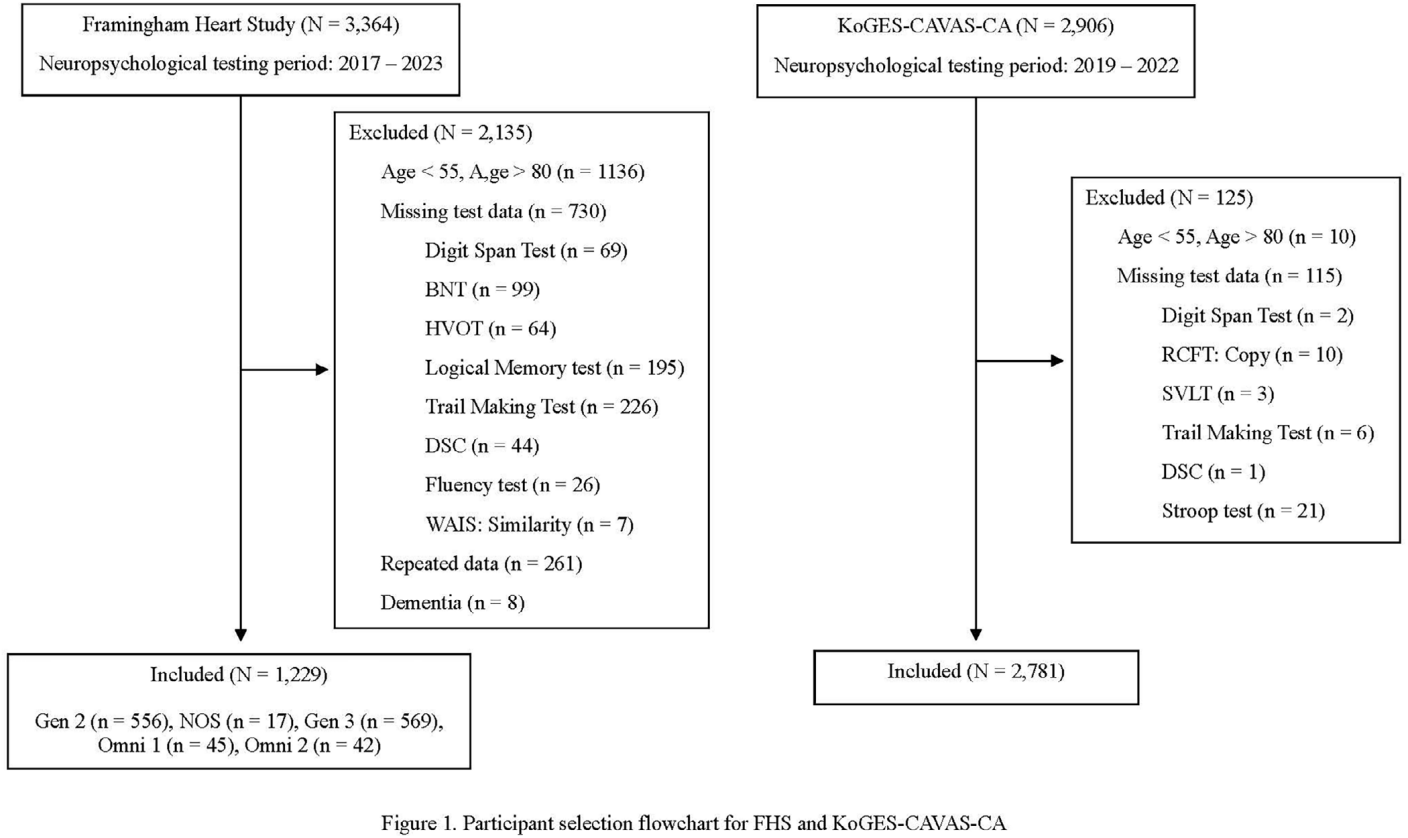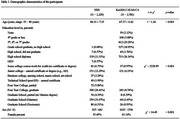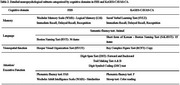# Cultural Effects of Demographic Factors on Cognitive Function: Findings from U.S. and Korea

**DOI:** 10.1002/alz70857_102885

**Published:** 2025-12-25

**Authors:** Minji Song, Ashita S. Gurnani, Katherine Gifford, Yu‐Mi Kim, Mi‐Kyung Kim, Mi‐Young Lee, Min‐Ho Shin, Sang‐Baek Koh, Hyeon‐Chang Kim, Yeonwook Kang, Rhoda Au

**Affiliations:** ^1^ Framingham Heart Study, Boston University Chobanian & Avedisian School of Medicine, Boston, MA, USA; ^2^ Boston University Chobanian & Avedisian School of Medicine, Boston, MA, USA; ^3^ Hanyang University, College of Medicine, Seoul, Korea, Republic of (South); ^4^ Hanyang University, Institute for Health and Society, Seoul, Korea, Republic of (South); ^5^ Keimyung University School of Medicine, Daegu, Korea, Republic of (South); ^6^ Chonnam National University Medical School, Gwangju, Korea, Republic of (South); ^7^ Wonju Severance Christian Hospital, Wonju College of Medicine, Wonju, Korea, Republic of (South); ^8^ Yonsei University, College of Medicine, Seoul, Korea, Republic of (South); ^9^ Hallym University, Chuncheon, Korea, Republic of (South); ^10^ Boston University School of Public Health, Boston, MA, USA

## Abstract

**Background:**

Cognitive functioning is influenced by demographic factors, and this relationship is further impacted by cultural differences. This study aimed to compare the effects of demographic factors on cognitive function in middle‐aged and older adults in the U.S. and Korea, providing insights into potential cultural influences.

**Method:**

A total of 1,229 individuals from the Framingham Heart Study (FHS) and 2,781 individuals from the Korean Genome and Epidemiology Study‐Cardiovascular Disease Association Study‐Cognitive Aging (KoGES‐CAVAS‐CA) were included (Figure 1, Table 1). The most recent neuropsychological tests of dementia‐free participants in each study were grouped into four cognitive domains: Memory, Language, Visuospatial Function (VF), and Attention/Executive Function (A/EF) (Table 2). Standardized domain scores were calculated using within‐sample z‐score standardization, and z‐tests assessed differences in demographic impact on cognitive function between the two populations.

**Result:**

FHS participants were older, more educated, with a higher proportion of men compared to KoGES‐CAVAS‐CA (Table 1). While Memory and A/EF were stable across ages in FHS, significant decline with increasing age was found in KoGES‐CAVAS‐CA (Memory: *z* = 12.61, *p* < 0.001; A/EF: *z* = 3.81, *p* < 0.001). Language declined with age in both populations, but the decline was significantly greater in KoGES‐CAVAS‐CA compared to FHS (*z* = 4.66, *p* < 0.001). Higher education was associated with better cognitive performance in both populations, but its effect on Language (*z* = ‐3.19, *p* = 0.001) and VF (*z* = ‐8.31, *p* < 0.001) was stronger in KoGES‐CAVAS‐CA than in FHS. Sex differences were more pronounced in Language in KoGES‐CAVAS‐CA compared to FHS, where women scored lower than men in both populations (*z* = 6.61, *p* < 0.001).

**Conclusion:**

These findings highlight cross‐cultural differences in how demographic factors influence cognitive function. The greater effect of education on Language and VF in KoGES‐CAVAS‐CA likely reflects differences in educational attainment between the two populations, with FHS having more college graduates and KoGES‐CAVAS‐CA participants averaging a middle school education. Additionally, more pronounced Language sex differences in KoGES‐CAVAS‐CA suggest sex‐related cognitive differences may vary across cultural contexts. These results emphasize the need to consider demographic disparities in cognitive research.